# Association of illness perception and alexithymia with fatigue in hemodialysis recipients: a single-center, cross-sectional study

**DOI:** 10.1038/s41598-023-43935-9

**Published:** 2023-10-03

**Authors:** Yoko Tanemoto, Ui Yamada, Masaaki Nakayama, Takeaki Takeuchi, Fumiaki Tanemoto, Yugo Ito, Daiki Kobayashi, Daisuke Ohta, Masahiro Hashizume

**Affiliations:** 1 Department of Psychosomatic Medicine, Toho University Graduate School of Medicine, Omori Nishi 5-21-16, Ota-Ku, Tokyo, 143-0015 Japan; 2 Department of Psychosomatic Medicine, St. Luke’s International Hospital, Akashicho 9-1, Chuo-Ku, Tokyo, 104-0044 Japan; 3 Department of Nephrology, St. Luke’s International Hospital, Akashicho 9-1, Chuo-Ku, Tokyo, 104-0044 Japan; 4 Department of Psychosomatic Medicine, Toho University School of Medicine, Omori Nishi 5-21-16, Ota-Ku, Tokyo, 143-0015 Japan; 5 Division of General Internal Medicine, Department of Internal Medicine, Tokyo Medical University Ibaraki Medical Center, Amicho Chuo 3-20-1, Inashiki-Gun, Ibaraki, 300-0332 Japan

**Keywords:** Psychology, Human behaviour, Quality of life, Fatigue, Renal replacement therapy, End-stage renal disease, Depression

## Abstract

Fatigue in hemodialysis recipients interferes with daily activities and renal rehabilitation, and its underlying causes and treatment remain unclear. Psychological factors, like illness perceptions and alexithymia, cause fatigue in other diseases; however, their contribution to hemodialysis-related fatigue is unknown. This cross-sectional study included 53 hemodialysis recipients. To assess participants’ fatigue, we used a self-administered patient-reported outcome questionnaire whose items have shown correlation with those of established scales, such as the Profile of Mood States and Visual Analogue Scales. The associations among the scores of the revised Illness Perceptions Questionnaire (IPQ-R), Toronto Alexithymia Scale (TAS-20), and Hospital Anxiety and Depression Scale and fatigue were analyzed using bivariable and multivariable analyses. Patients with fatigue had significantly higher median scores for the IPQ-R subscales “Identity” and “Negative emotional representation about illness” than those without fatigue, suggesting the association of specific illness perception with fatigue. Median scores for the TAS-20 subscale “Difficulty identifying feelings” were also significantly higher among fatigued patients, suggesting the association of alexithymia with fatigue. Depression was not associated with fatigue. Multivariable logistic regression revealed the association of a high “Identity” score with the risk of fatigue (adjusted odds ratio, 1.32; 95% confidence interval, 1.00–1.73; P = 0.04), while there were no significant association between a high “Difficulty identifying feelings” score and the risk of fatigue (adjusted odds ratio, 1.09; 95% confidence interval, 0.95–1.24). Specific illness perception and alexithymia were slightly associated with hemodialysis-related fatigue. Cognitive-behavioral therapy for these conditions could reduce fatigue and promote renal rehabilitation.

## Introduction

Fatigue is an extremely common symptom among hemodialysis recipients; the prevalence has been reported previously as 60–97%^1^. It not only is associated with quality of life (QoL)^2^, but also affects physical and social roles in daily lives^3^. According to patients on hemodialysis, fatigue is one of the major symptoms for which new therapies should be developed^4^.

Causes of fatigue in hemodialysis recipients are complex^1^. Physiological and hemodialysis-related factors can be modified to some extent. In contrast, no treatment targeting psychological factors that contribute to fatigue has yet been established, although hemodialysis recipients have many known stressors^5^. Depression is statistically associated with hemodialysis-related fatigue, according to several studies^6^; however, treatment for depression has not been shown to reduce fatigue in these patients. In this study, we focused on the variability in individual patients’ perceptions of stress that underlie the development of symptoms and coping behaviors.

**Figure 1:**
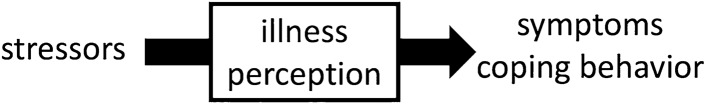
The role of illness perception. Illness perception is the process of perceiving illness-related stress that leads to symptoms and shapes coping behavior.

Perception of stress is extremely critical because it can modify symptoms and shape coping behaviors. In particular, perception of stress related with chronic diseases is based on patients’ beliefs and knowledge of their condition, which is called as “illness perception”^7^. Illness perception is originally described as a part of the theoretical basis in Leventhal’s Self-regulatory Model, which outlines how illness gives rise to patients' responses which in turn determine their coping behaviors. Patients develop symptoms and cope with them through a process of judging the extent of their illness and associated problems (stressors) by their own illness perception (Fig. 1). In the Self-regulatory Model, Leventhal proposed five key factors: cause, identity, timeline, control, and consequences. Later, two dimensions of emotional representations and illness coherence were added to them. According to these dimensions, researchers have explored illness perceptions in patients with fatigue in previous studies. For example, adolescents with chronic fatigue syndrome were shown to differ from those with other diseases, such as type 1 diabetes and juvenile idiopathic arthritis, in terms of the consequences, timeline, control, and identity^8^, and cancer survivors with more fatigue had higher scores for consequences and identity than those with less fatigue^9^. Another study suggested that stronger beliefs in identity, timeline, and consequences were associated with more severe fatigue, and better control was associated with lower degrees of fatigue in patients with multiple sclerosis^10^.

A type of inability called alexithymia may also play a role in the formation of patients’ perception of stress. Alexithymia was originally defined as a core characteristic of patients with psychosomatic diseases and has been developed as a concept reflecting an inability in the cognitive processing and regulation of emotions^11^. As people with alexithymia have difficulty in identifying and describing their and others’ emotions, they tend to attribute adverse circumstances to external causes and not their emotions. Such people are prone to somatization^11^ (i.e., converting the suffering of stress to physical symptoms). In patients with multiple sclerosis^12^ and cancer^13^, previous studies have suggested that alexithymia contributes to fatigue.

To our knowledge, no previous study has focused on the role of illness perception or alexithymia in fatigue in hemodialysis recipients. To address this gap, we designed this cross-sectional study at a dialysis treatment center in a Japanese community hospital to examine whether specific illness perception or alexithymia is associated with fatigue in hemodialysis recipients.

## Materials and methods

### Study design and participants

For this cross-sectional study, 81 of 90 patients receiving maintenance hemodialysis at the dialysis center of St. Luke’s International Hospital, Tokyo, Japan, were recruited according to the following criteria between November 2019 and October 2020. Selection criteria were age of ≥ 20 years and receiving hemodialysis for ≥ 3 months. Furthermore, we excluded patients who received hemodialysis in combination with peritoneal dialysis and those who were unable to consent to participate and thus, despite assistance, unable to answer questionnaires for any reason. All protocols in this study were approved by the Ethics Committee of St. Luke’s International Hospital (IRB approval number 19-R060). All procedures in this study were performed in accordance with the ethical standards of the Ethics Committee of St. Luke’s International Hospital and with the 1964 Helsinki declaration and its later amendments or comparable ethical standards including the Japanese government’s Ethical Guidelines for Medical and Health Research Involving Human Subjects. Informed consent was obtained from all participants included in the study.

### Measurement tools

#### Measurement of fatigue

To assess participants’ fatigue, we used a self-administered modified patient-reported outcome questionnaire (Table S1) whose items have shown correlation with those of established scales, such as the Profile of Mood States and Visual Analogue Scales^14^. Fatigue was categorized as grade 0 (tireless; behaving normally without a sense of fatigue), grade 1 (mild fatigue; behaving normally but feeling tired), grade 2 (moderate fatigue; feeling tired with light work), and grade 3 (intense fatigue; becoming very tired and falling asleep after light work). Patients who chose grades 1, 2 and 3 fatigue were included in the “fatigue group,” and those who chose grade 0 fatigue, in the “non-fatigue group.”

#### Measurement of illness perception

Illness perception is a series of perceptions about a patient’s illness, characterized by the cause (what the patient thinks are causes of the illness) and the levels of the following dimensions: identity (the number of subjective physical symptoms that the patient attributes to the illness), timeline (whether the patient thinks the illness is acute, chronic, or cyclical), consequences (how the patient thinks about the effect of the illness on his or her life), personal/treatment control (how the patient thinks about how much he or she can do to ameliorate the illness), emotional representation (the patient’s negative/positive emotional response to the illness/treatment)*,* and illness coherence (the sense of having a comprehensive understanding of the illness). The revised Illness Perception Questionnaire (IPQ-R) is a measure of each dimension just described^15^. We used the Japanese version of the IPQ-R, which has proven validity and reliability; Chronbach’s α for each subscale showed internal consistency (α = 0.68 to 0.93) except for α = 0.52 for the subscale “illness coherence” and the Pearson correlations for test–retest reliability varied between 0.42 and 0.86^16^. Each item is rated on a 5-point Likert scale.

#### Assessment of alexithymia

Alexithymia is represented as three dimensions: difficulty identifying feelings, difficulty describing feelings, and externally oriented thinking. The Toronto Alexithymia Scale (TAS-20)^17^ is used to assess each score of those dimensions and the total score; higher scores indicate alexithymia. In this study, we used the validated Japanese version of the TAS-20; Chronbach’s α for each subscale score and the total score showed internal consistency (α = 0.66 to 0.85) except for α = 0.48 to 0.58 for the subscale “Externally oriented thinking” and the Pearson correlations for test–retest reliability varied between 0.626 and 0.757^18^*.* Each item is graded on a 5-point Likert scale.

#### Measurements of depression and anxiety

The Hospital Anxiety and Depression Scale (HADS) is a reliable instrument for detecting and measuring levels of depression and anxiety states in patients at medical outpatient clinics^19^. We used the Japanese version of the HADS^20^, which has been validated in a previous study; Chronbach’s α for each subscale showed internal consistency (α = 0.75 to 0.87) and the Pearson correlations for test–retest reliability varied between 0.46 and 0.72^21^. Each item is rated on a 4-point Likert scale. Higher scores of each subscale (depression and anxiety), especially 8 and more out of 0 to 21, are indicative of depression and anxiety.

#### Other assessments

Information about patient demographics (age and sex), clinical information about hemodialysis (duration, interdialytic weight gain, and presence of intradialytic hypotension), and biochemical data (hemoglobin, albumin, and C-reactive protein levels) were collected from the St. Luke’s International Hospital’s database. Blood samples were obtained at the start of regular hemodialysis treatment on Mondays or Tuesdays. Average values for a 3-month period for interdialytic weight gain and each biochemical parameter were obtained in the assessments. The presence of intradialytic hypotension was defined as an acute decline in systolic blood pressure to < 100 mmHg during dialysis at least once in the 3-month period. To measure QoL, we used the Japanese version of the Kidney Disease Quality of Life—Short Form (KDQOL-SF)^22^.

### Statistical analyses

We used EZR (Saitama Medical Center, Jichi Medical University, Saitama, Japan)^23^, a graphical user interface for R software (The R Foundation for Statistical Computing), to perform all statistical analyses. Categorical variables were calculated as numbers with percentages, and continuous variables, as medians with overall ranges. The Mann–Whitney *U* test and Fisher's exact test were used to compare baseline clinical characteristics of the fatigue group with those of the non-fatigue group. To analyze the relationships between fatigue and other clinical measures (illness perception, alexithymia, depression, and anxiety), we used the Mann–Whitney *U* test. The associations between the measures of illness perception or alexithymia and fatigue were examined with bivariable analyses, and multivariable logistic regression analyses. The effect size values were expressed as Cliff’s delta (calculated by The R Base Package 4.3.1, effsize 0.8.1). Multivariable logistic regression analyses were used to show the effects of the covariables on the outcome measure after adjustment for other covariables (age, sex, duration of dialysis, and overall health rating score on the KDQOL-SF). These covariates were selected a priori as potential confounders. Separate models for each subscale of illness perception and alexithymia were run in logistic regression.

## Results

Of the 81 patients recruited at the dialysis center of St. Luke’s International Hospital, 53 met the entry criteria and completed the questionnaires. All of them were mongoloid and 79% of them were male. 29 were classified as the fatigue group and the rest as the non-fatigue group. No significant differences were found in the demographic, clinical, and biochemical characteristics between the fatigue and non-fatigue groups. The overall health rating score was significantly lower in the fatigue group (Table 1).

**Table 1:** Characteristics of the participants.

Characteristic		Total (*n* = 53 [100%])	Fatigue group (*n* = 29 [54.7%])	Non-Fatigue group (*n* = 24 [45.2%])	*P*-value
Age, years		67 (36–87)	67 (36–87)	65 (47–87)	0.83
Male		42 (79%)	20 (68%)	22 (91%)	0.08^a^
Fatigue					
Grade 0		24 (45%)	–	24 (100%)	–
Grade 1		19 (35%)	19 (65%)	–	–
Grade 2		8 (15%)	8 (27%)	–	–
Grade 3		2 (3%)	2 (6%)	–	–
Duration of hemodialysis, years		7 (0–30)	4 (0–30)	7 (0–22)	0.40
Interdialytic weight gain, kg		2.7 (0.5–5.2)	2.8 (0.5–4.7)	2.4 (0.7–5.2)	0.57
Intradialytic hypotension		40 (75%)	23 (79%)	17 (70%)	0.53^a^
Diabetes mellitus		27 (50%)	14 (48%)	13 (54%)	0.78^a^
Cardiovascular disease		15 (28%)	8 (27%)	7 (29%)	1.00^a^
Current malignancy		2 (3%)	1 (3%)	1 (4%)	1.00^a^
Hemoglobin level, g/dL		10.8 (8.2–14.2)	10.8 (8.2–12.9)	11.1 (9.1–14.2)	0.51
Albumin level, g/dL		3.8 (2.4–4.3)	3.7 (2.9–4.3)	3.8 (2.4–4.1)	0.23
C-reactive protein level, mg/L		0.12 (0.01–3.62)	0.14 (0.03–3.24)	0.10 (0.01–3.62)	0.24
KDQOL-SF					
Overall health rating		69 (31–89)	64 (40–85)	76 (31–89)	0.02*

Data are expressed as medians (with overall ranges) or as numerical values (with percentages).

*P < .05.

^a^Assessed with Fisher’s exact test. The other characteristics were assessed with the Mann–Whitney *U* test.

### Some dimensions of illness perception were associated with fatigue in patients on hemodialysis

To find the specific pattern of illness perception of hemodialysis recipients with fatigue, we compared the levels of each IPQ-R dimension of illness perception in the fatigue and non-fatigue groups. Median scores on the subscales “Identity” (P < 0.01, Cliff’s delta effect size = 0.482) and “Negative emotional representation about illness” (P < 0.05, Cliff’s delta effect size = 0.386) were significantly higher in the fatigue group than in the non-fatigue group. With regard to each item of causes, scores on the “Causes—stress or worry” (P < 0.05, Cliff’s delta effect size = 0.330) and “Causes—poor medical care in my past” (P < 0.05, Cliff’s delta effect size = 0.303) were significantly higher in the fatigue group than in the non-fatigue group (Table 2).

**Table 2:** IPQ-R (illness perception) scores by group.

Subscale (score range)	Fatigue group (*n* = 29)	Non-Fatigue group (*n* = 24)	*P*-value^a^	Cliff’s Delta
Identity (0–14)	6 (2–13)	3 (0–12)	0.002**	0.482
Timeline—acute/chronic (6–30)	23 (17–25)	25 (17–25)	0.93	0.056
Consequence (6–30)	20 (15–29)	21 (16–30)	0.73	0.053
Personal control (6–30)	25 (11–32)	24 (19–35)	0.82	0.034
Treatment control (6–30)	17 (5–23)	17 (11–23)	0.64	0.073
Illness coherence (5–25)	18 (13–25)	19 (12–25)	0.35	0.147
Timeline—cyclical (4–20)	10 (4–15)	10 (4–14)	0.84	0.030
Negative emotional representation about illness (6–30)	18 (10–29)	14 (8–30)	0.01*	0.386
Positive emotional representation about illness (4–20)	6 (4–12)	6 (4–10)	0.61	0.079
Negative emotional representation about treatment (6–30)	18 (8–27)	15 (10–25)	0.16	0.219
Positive emotional representation about treatment (4–20)	11 (4–17)	11 (4–16)	0.45	0.117
Causes^b^				
Stress or worry (1–5)	3 (1–5)	2 (1–5)	0.03*	0.330
Hereditary—it runs in my family (1–5)	3 (1–5)	2 (1–5)	0.97	0.005
A germ or virus (1–5)	2 (1–4)	2 (1–5)	0.72	0.053
Diet or eating habits (1–5)	4 (1–5)	4 (1–5)	0.68	0.060
Chance or bad luck (1–5)	3 (1–5)	2 (1–5)	0.1	0.255
Poor medical care in my past (1–5)	3 (1–5)	2 (1–5)	0.04*	0.303
Pollution in the environment (1–5)	2 (1–4)	1 (1–3)	0.13	0.224
My own behavior (1–5)	4 (1–5)	3 (1–5)	0.51	0.102
My mental attitude; e.g., thinking about life negatively (1–5)	2 (1–4)	2 (1–5)	0.76	0.045
Family problems or worries caused my illness (1–5)	2 (1–4)	2 (1–5)	0.44	0.109
Overwork (1–5)	3 (1–5)	3 (1–5)	0.94	0.011
My emotional state; e.g., feeling down, lonely, anxious, empty (1–5)	2 (1–5)	2 (1–5)	0.87	0.024
Aging (1–5)	3 (1–5)	3 (1–5)	0.64	0.073
Alcohol (1–5)	2 (1–5)	2 (1–5)	0.61	0.079
Smoking (1–5)	2 (1–5)	2 (1–5)	0.72	0.054
Accident or injury (1–5)	1 (1–3)	1 (1–3)	1.00	0
My personality (1–5)	3 (1–5)	2 (1–4)	0.25	0.178
Altered immunity (1–5)	3 (1–4)	2 (1–5)	0.37	0.135

Data are expressed as medians (with overall ranges).

*P < .05. **P < .01.

^a^Mann–Whitney *U* test.

^b^Each cause is rated on a scale of 1 to 5.

### Alexithymia was associated with fatigue in patients on hemodialysis

To find differences in terms of alexithymia between the fatigue and non-fatigue groups, we measured each level of the TAS-20 dimensions of alexithymia in those groups. The score on the subscale “Difficulty identifying feelings” (P < 0.05, Cliff’s delta effect size = 0.369) was significantly higher in the fatigue group than in the non-fatigue group (Table 3).

**Table 3:** TAS-20 (alexithymia) scores by group.

	Fatigue group (*n* = 29)	Non-Fatigue group (*n* = 24)	*P*-value^a^	Cliff’s Delta
TAS-20 total score (20–100)	48 (30–64)	45 (33–64)	0.25	0.182
Scores ≧ 52^b^, *n* (%)	11 (37%)	7 (29%)	0.56	0.087
Subscale (score range)				
Difficulty identifying feelings (7–35)	15 (7–28)	11 (7–23)	0.02*	0.369
Difficulty describing feelings (5–25)	14 (5–21)	13 (6–20)	0.78	0.043
Externally oriented thinking (8–40)	21 (14–27)	21 (12–29)	0.36	0.145

Except where noted, data are expressed as medians (with overall ranges) of subscale scores.

*TAS-20* Toronto Alexithymia Scale.

*P < .05.

^a^Mann–Whitney *U* test.

^b^A total score of 52 or higher indicates possible alexithymia.

### Depression and anxiety were not associated with fatigue in patients on hemodialysis

Depression and anxiety have been established as factors contributing to fatigue in previous studies of hemodialysis recipients. We assessed the participants’ depression and anxiety levels by using the HADS. Depression and anxiety scores of patients with fatigue did not differ significantly from those of patients without fatigue. Also, the proportions of patients whose depression and anxiety scores were above the cutoff score of 8 did not differ significantly between the fatigue and non-fatigue groups (Table 4).

**Table 4:** HADS (depression and anxiety) scores by group.

Subscale (score range)	Fatigue group (*n* = 29)	Non-Fatigue group (*n* = 24)	*P*-value^a^	Cliff’s delta
Depression (0–21)	5 (1–18)	4 (0–15)	0.41	0.132
Score ≧ 8, *n* (%)^b^	10 (34%)	8 (33%)	1.00	0.011
Anxiety (0–21)	2 (0–15)	1 (0–14)	0.88	0.024
Score ≧ 8, *n* (%)^b^	6 (20%)	5 (20%)	1.00	0.001

Data are expressed as medians (with overall ranges) or as numerical values (with percentages).

*HADS* Hospital Anxiety and Depression Scale.

^a^The scores on the “Depression” and “Anxiety” subscales were evaluated through the Mann–Whitney *U* test; the numbers of patients suspected of depression or anxiety were assessed through Fisher’s exact test.

^b^For each subscale, a score of 8 or higher indicates depression or anxiety.

### Illness perception was an independent contributor to fatigue in patients on hemodialysis

Finally, we conducted logistic regression analyses, adjusted for patients’ age, sex, duration of dialysis and QoL (the score of overall health on the KDQOL-SF), to check the incidence of fatigue. High scores on the IPQ-R subscale “Identity” were associated with a risk of fatigue (adjusted odds ratio, 1.32; 95% confidence interval, 1.00–1.73; P = 0.04; Table 5).

**Table 5:** Odds ratios and 95% confidence intervals for the association between fatigue and illness perception/alexithymia.

Variables	Crude OR	95% CI	Model 1^a^		Model 2^b^	
			Adjusted OR	95% CI	Adjusted OR	95% CI
IPQ-R (illness perception)						
Identity	1.33	1.09–1.62**	1.37	1.11–1.68**	1.32	1.00–1.73*
Negative emotional representation about illness	1.13	1.01–1.27*	1.13	1.01–1.27*	1.08	0.94–1.22
Causes						
Stress or worry	1.71	1.03–2.83*	1.72	1.02–2.89*	1.55	0.89–2.68
Poor medical care in my past	1.71	0.97–3.01	1.82	0.99–3.31	1.72	0.91–3.23
TAS-20 (alexithymia)						
Difficulty identifying feelings	1.14	1.01–1.28*	1.14	1.01–1.29*	1.09	0.95–1.24

*OR* odds ratio, *CI* confidence interval.

*P < .05 (multivariable logistic regression model).

**P < .01 (multivariable logistic regression model).

^a^Adjusted for age and duration of dialysis.

^b^Adjusted for age, duration of dialysis, male, and the overall health rating score of the Kidney Disease Quality of Life—Short Form.

## Discussion

**Figure 2:**
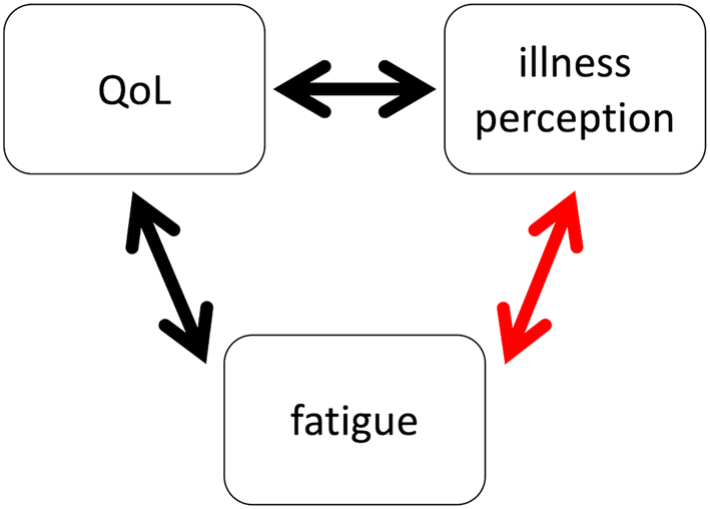
Known associations between fatigue and quality of life (QoL) and between QoL and illness perception in patients undergoing hemodialysis. In such patients, fatigue is known to be associated with QoL and QoL is known to be associated with illness perception. This study has newly shown that illness perception is a contributor to hemodialysis patients’ fatigue independently of QoL.

We demonstrated that specific illness perceptions and alexithymia were associated with fatigue in hemodialysis recipients. We found no significant differences in demographic, hemodialysis-related, and physiological characteristics between the fatigue and non-fatigue groups, while overall QoL was poorer in fatigue group (Table 1), especially in terms of symptoms and physical role (effect of illness on physical function) (Table S2); this finding was consistent with that in a previous report^2^. As for illness perception, the results showed that hemodialysis recipients with higher scores on the IPQ-R subscale “Identity” (attributing more symptoms to chronic kidney disease [CKD]) were more often fatigued, regardless of age, sex, duration of dialysis, and QoL (Table 5). Although the associations between fatigue and QoL ^2^ and between QoL and illness perception^24^ in hemodialysis recipients have already been demonstrated, a new finding in this study was that illness perception is an independent contributor to fatigue in such patients (Fig. 2). Moreover, hemodialysis recipients with fatigue might tend to feel stress, to be worried, and to blame poor (as perceived by the patient) past medical care for the causes of CKD (Table 2). With regard to alexithymia, it was observed that hemodialysis recipients with fatigue had greater difficulty in identifying feelings (Table 3). Notably, the results did not show associations between depression and fatigue (Table 4).

Among psychological factors related to fatigue in hemodialysis recipients, depression has been the most extensively studied so far. Although several studies have shown statistically significant correlations between fatigue and depression^6^, interestingly no depression-targeted treatment, such as selective serotonin reuptake inhibitors^25^, has been proven to be an effective treatment for reducing dialysis-related fatigue. In this regard, depression might not be all the cause and may be one of the results of hemodialysis recipients’ perception of stress. Actually, we could not observe any association between fatigue and depression in hemodialysis recipients. One reason for the inconsistency in past studies might be the difference in evaluation methods of depression; HADS, used in our study, is made for assessment of patients with physical illness and focuses mainly on nonphysical symptoms so that it has few items influenced by physical illness, whereas the Beck Depression Inventory and Patient Health Questionnaire, which were used in the majority of previous studies^6^, contain questions about physical symptoms, including fatigue, which could lead to overestimation. In addition, difference of national characteristics could be another reason; most of our study subjects were Japanese, and there may be a cultural background about stigma towards depression that makes them tend not to report depression according to previous studies^26–28^. This issue could be tested by using methods other than self-administered questionnaires, ideally structured interviews. Moreover, previous reports have shown a lower prevalence of depression in Japan than in other countries, which may be explained by differences in not only cultural factors but also genetic factors and cross-cultural application of diagnostic criteria^29^. Therefore, we conducted additional analyses targeting patients not suspected of having depression (those with HADS “Depression” subscale scores of < 8; Tables S3 and S4) and confirmed that high scores for the IPQ-R subscales “Identity,” “Negative emotional representation about illness,” and “Causes—stress or worry” were still associated with fatigue. This result may suggest that other factors besides depression are related to fatigue in hemodialysis recipients.

Illness perception has been revealed as one of the key contributors to fatigue in patients with certain other diseases, such as adolescents with chronic fatigue syndrome, cancer survivors, and patients with multiple sclerosis^8–10^. Across these studies, illness perceptions contributing to fatigue seemed to differ among patients with different diseases. To our knowledge, this is the first study that may suggest specific illness perceptions that contribute to fatigue in hemodialysis recipients. We reveal that hemodialysis recipients with fatigue tend to have negative emotional responses to chronic kidney disease, whether they feel depressed or not, and attribute more physical symptoms, stress or worry, and previous medical care toward CKD. These characteristics of illness perception may be affected by stressors that are specific to hemodialysis recipients, who have been overwhelmed by the whole experience of having been diagnosed with CKD to be ESKD and being on hemodialysis now.

Furthermore, alexithymia, which is known to be conducive to somatization^11^ has been suggested to be associated with specific physical symptom and their severity. It is reported to be a possible contributor to fatigue in patients with diseases such as multiple sclerosis and cancer^12,13^. Our study, too, revealed difficulty in identifying feelings, a characteristic of alexithymia, in fatigued patients on hemodialysis. We suggest that hemodialysis-related fatigue may be a result of the somatization of stress in hemodialysis recipients.

**Figure 3:**
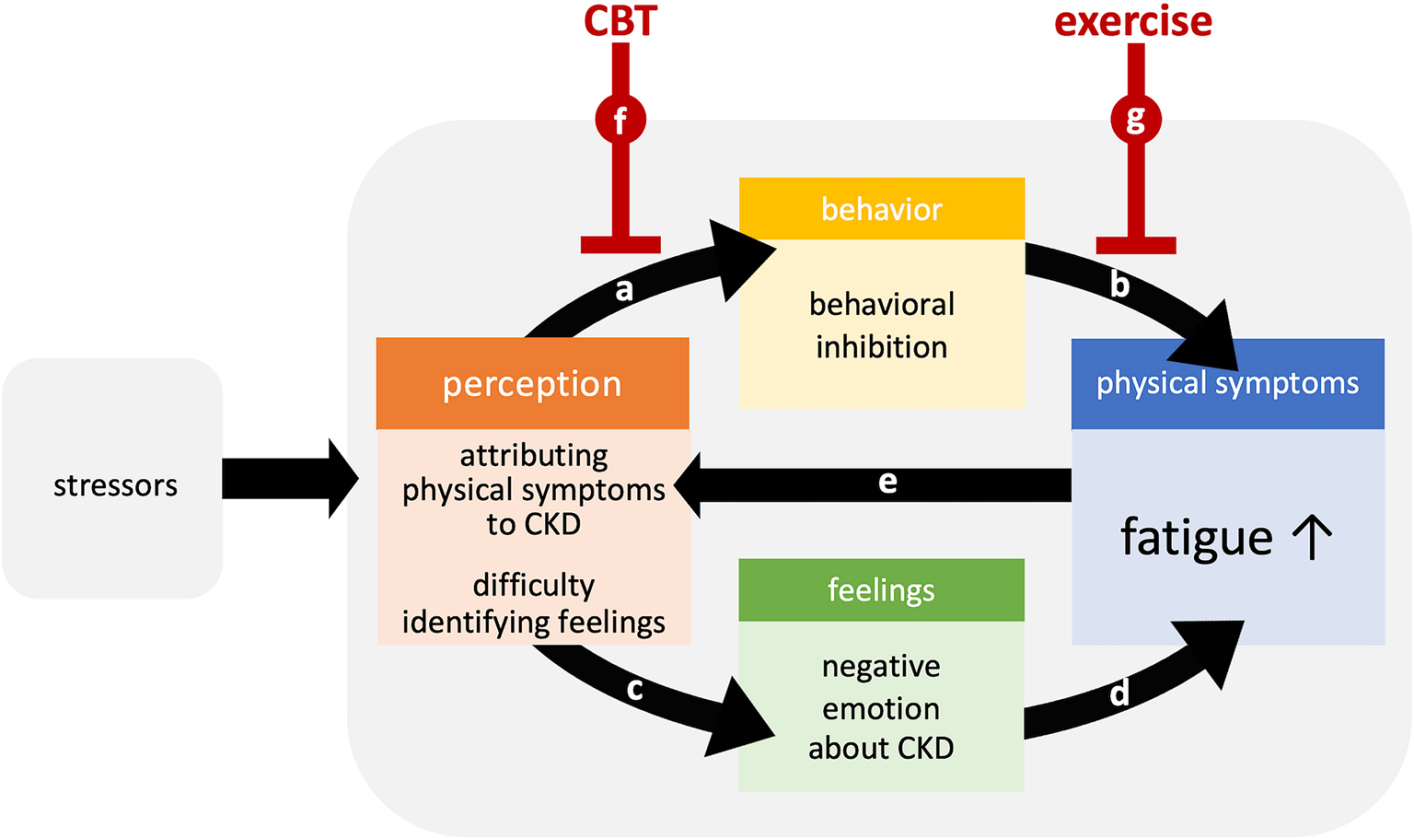
Possible therapeutic targets in the cognitive–behavioral model of hemodialysis-related fatigue based on our working hypothesis. Hemodialysis recipients with fatigue may tend to attribute their physical symptoms to chronic kidney disease (CKD), which causes them to restrict their own role in daily life (“behavioral inhibition”) (**a**), which in turn exacerbates fatigue (**b**). Moreover, they may tend to have negative emotions about CKD, however patients who have alexithymia do not identify their emotions (**c**), which are expressed as further physical symptoms, including fatigue (**d**). This reinforces their perception that CKD causes worsening of fatigue (**e**). Cognitive–behavioral therapy (CBT) is a possible intervention targeting their perception, which causes behavioral inhibition constituting this vicious circle (**f**). Exercise would help to break this pattern, approaching their behavioral inhibition (**g**). Renal rehabilitation, in which exercise therapy is the main focus, could be more effective when combined with CBT.

In Fig. 3, we present a cognitive–behavioral model of the pathogenesis of fatigue in hemodialysis recipients based on our working hypothesis of the present study. Hemodialysis recipients with fatigue tend to attribute their physical symptoms to CKD and consequently restrict their own roles in daily life. This “behavioral inhibition,” such as lack of exercise (which exacerbates fatigue), was shown to be associated with the severity of dialysis-related fatigue^30^. Furthermore, hemodialysis recipients with fatigue tend to have negative emotions about CKD as a result of their own physical symptoms, but in those with alexithymia, their emotions are not identified and instead are expressed as additional physical symptoms, including fatigue. Consequently, such patients consider their CKD more severe because of worsened fatigue. This vicious circle could lead to the maintenance and worsening of fatigue in hemodialysis recipients (Fig. 3). Approaches targeting this perception pattern, such as perception reframing (a kind of cognitive–behavioral therapy [CBT]), could be beneficial in breaking that circle and reducing their fatigue. In fact, CBT is an effective treatment that reduces fatigue in several diseases^31–33^.

Moreover, this model explains why exercise is not well practiced by fatigued hemodialysis recipients, although they do regard exercise as beneficial in reducing their fatigue^34,35^. According to a recent report, exercise during renal rehabilitation is highly recommended for the patients who underwent hemodialysis as it improved exercise tolerance, walking ability, and physical QOL^36^. However, the patients’ perceptions of their symptoms might cause them to avoid exercise, which results in aggravated fatigue and reduces their hope of maintaining their physical and social roles while performing daily lives. CBT skills specific to fatigue-related perception by instructors of renal rehabilitation could be the key factor for increasing participation in exercise and promoting awareness regarding renal rehabilitation (Fig. 3).

This study had several limitations. First, it was a single-center study in a community hospital in Tokyo, Japan; thus because the findings could reflect trends specific to Japanese urban people, including the profile of the depressive state, they might not be fully generalizable. However, perception patterns obviously differ between patients with and without fatigue who receive the same treatment and care under similar circumstances. In addition, because of the small number of subsets, the confidence intervals became large. Second, because we studied associations between fatigue and several participant characteristics, some associations may have been falsely positive. It needs further investigations to confirm the reproducibility of the present findings. Finally, this was a cross-sectional study whose results were limited to associations, not causality. Future intervention trials, such as CBT, are needed to confirm our results. Despite these limitations, this was a unique study in that we compared illness perceptions and alexithymia of hemodialysis recipients with and without fatigue.

In conclusion, our results indicate that illness perception and alexithymia of hemodialysis recipients could affect their fatigue. Fatigue in such patients may be associated both with alexithymia and with an illness perception in which they attribute physical symptoms to the illness. CBT for these conditions could reduce fatigue and promote the spread of renal rehabilitation; further intervention trials are needed to validate our finding of perception of stress as a therapeutic target.

### Supplementary Information

Supplementary Tables.

## Data Availability

The data of this article cannot be shared publicly to protect the privacy of individuals who participated in the study. The data will be shared upon reasonable request to the corresponding author.
