# Supplementary material for: Association of illness perception and alexithymia with fatigue in hemodialysis recipients: a single-center, cross-sectional study
Source: Sci Rep. 2023 Oct 3;13:16592. doi: 10.1038/s41598-023-43935-9 (PMC10547682; doi:10.1038/s41598-023-43935-9)
Supplement: Supplementary file 1 — Supplementary Tables. [file 41598_2023_43935_MOESM1_ESM.pdf]

Title

Association of illness perception and alexithymia with fatigue in hemodialysis recipients: A single-center, cross-sectional study

The names of the authors

Yoko Tanemoto, M.D.[1,2], Ui Yamada, M.D., Ph.D.[2], Masaaki Nakayama, M.D., Ph.D.[3], Takeaki Takeuchi, M.D., Ph.D.[4], Fumiaki Tanemoto, M.D.[3], Yugo Ito, M.D., Ph.D.[3], Daiki Kobayashi, M.D., Ph.D.[5], Daisuke Ohta, M.D., Ph.D.[2], Masahiro Hashizume, M.D., Ph.D.[1,4]

The affiliations and addresses of the authors

[1] Department of Psychosomatic Medicine, Toho University Graduate School of Medicine, Omori nishi 5-21-16, Ota-ku, Tokyo, [2] Department of Psychosomatic Medicine, St. Luke's International Hospital, Akashicho 9-1, Chuo-ku, Tokyo, [3] Department of Nephrology, St. Luke's International Hospital, Akashicho 9-1, Chuo-ku, Tokyo, [4] Department of Psychosomatic Medicine, Toho University School of Medicine, Omori nishi 5-21-16, Ota-ku, Tokyo, [5] Division of General Internal Medicine, Department of Internal Medicine, Tokyo Medical University Ibaraki Medical Center, Amicho chuo 3-20-1, Inashiki-gun, Ibaraki

The e-mail address of the corresponding author

Yoko Tanemoto

E-mail address: yokoichi@luke.ac.jp

Supplementary material

Table S1. The original questionnaire and the modified questionnaire to measure fatigue

(a) The original questionnaire to measure fatigue

grade	
1	inexhaustible
2	tireless
3	mild fatigue
4	moderate fatigue
5	intense fatigue

(b) The modified questionnaire to measure fatigue

grade	
0	tireless
1	mild fatigue
2	moderate fatigue
3	intense fatigue

Table S2. KDQOL-SF scores by group

KDQOL item	Fatigue group (*n* = 29)	Non-Fatigue group (*n* = 24)	*P*-value[a]
**Overall health rating (0–100)**	64 (40–85)	76 (31–89)	.02*
**Kidney disease-targeted scales (0–100)**			
**Symptom/problems**	83 (59–97)	89 (62–100)	.03*
**Effects of kidney disease**	78 (59–93)	84 (37–100)	.11
**Burden of kidney disease**	37 (0–81)	43 (0–81)	.42
**Work status**	50 (0–100)	50 (0–100)	.35
**Cognitive function**	86 (46–100)	93 (6–100)	.22
**Quality of social interaction**	86 (46–100)	93 (13–100)	.05
**Sexual function**	NA	NA	NA
**Sleep**	62 (20–97)	72 (22–100)	.06
**Social support**	66 (0–100)	66 (49–100)	.18
**Dialysis staff encouragement**	75 (50–100)	75 (50–100)	.19
**Patient satisfaction**	83 (33–100)	83 (50–100)	.52
**36-item health survey scales (0–100)**			
**Physical functioning**	65 (15–95)	80 (0–100)	.21
**Role—physical**	50 (0–100)	100 (0–100)	.03*
**Pain**	67 (12–100)	85 (20–100)	.05
**General health perceptions**	40 (20–75)	50 (10–80)	.07
**Energy/fatigue**	55 (20–85)	57 (0–100)	.24
**Social function**	75 (12–100)	87 (50–100)	.31
**Role—emotional**	66 (0–100)	100 (0–100)	.11
**Emotional well-being**	72 (28–96)	78 (0–100)	.22

Data are expressed as medians (with overall ranges).

*$P < .05$

[a]Mann–Whitney *U* test.

KDQOL-SF, Kidney Disease Quality of Life—Short Form. Role—physical, effect of illness on physical function. Role—emotional, effect of illness on emotional function.

Table S3. IPQ (illness perception) scores by group (score of <8 on HADS "Depression" subscale)

Subscale and causes and score ranges	Fatigue group (n = 19)	Non-Fatigue group (n = 16)	P-value[a]
**Identity (0–14)**	6 (2–13)	1 (0–12)	.001*
**Timeline (6–30)**	25 (20–25)	24 (18–25)	.50
**Consequence (6–30)**	20 (15–26)	20 (16–30)	.85
**Personal control (6–30)**	26 (17–31)	24 (21–34)	.46
**Treatment control (6–30)**	17 (9–23)	17 (12–23)	.27
**Illness coherence (5–25)**	19 (13–25)	19 (12–25)	.40
**Cyclical timeline (4–20)**	9 (4–14)	10 (4–14)	.97
**Negative emotional representation about illness (6–30)**	18 (10–29)	13 (8–30)	.008*
**Positive emotional representation about illness (4–20)**	6 (4–12)	7 (4–10)	.85
**Negative emotional representation about treatment (6–30)**	18 (8–27)	15 (11–24)	.12
**Positive emotional representation about treatment (4–20)**	10 (4–14)	11 (10–16)	.07
**Causes**			
**Stress or worry (1–5)**	4 (1–5)	2 (1–4)	.003*
**Hereditary—it runs in my family (1–5)**	2 (1–5)	3 (1–5)	.46
**A germ or virus (1–5)**	2 (1–4)	2 (1–3)	.21
**Diet or eating habits (1–5)**	4 (1–5)	4 (1–5)	.15
**Chance or bad luck (1–5)**	2 (1–5)	2 (1–5)	.87
**Poor medical care in my past (1–5)**	3 (1–5)	2 (1–3)	.08
**Pollution in the environment (1–5)**	2 (1–4)	1 (1–3)	.31
**My own behavior (1–5)**	4 (1–5)	3 (1–5)	.22
**My mental attitude; e.g., thinking about life negatively (1–5)**	2 (1–4)	2 (1–4)	.84

**Family problems or worries caused my illness (1–5)**	2 (1–4)	2 (1–3)	.66
**Overwork (1–5)**	3 (1–5)	2 (1–4)	.13
**My emotional state**; **e.g.**, **feeling down, lonely, anxious, empty (1–5)**	2 (1–5)	2 (1–4)	.68
**Aging (1–5)**	3 (1–5)	3 (1–4)	1.00
**Alcohol (1–5)**	3 (1–5)	2 (1–5)	.39
**Smoking (1–5)**	2 (1–4)	2 (1–5)	.61
**Accident or injury (1–5)**	1 (1–3)	1 (1–3)	.80
**My personality (1–5)**	3 (1–5)	2 (1–4)	.07
**Altered immunity (1–5)**	3 (1–4)	2 (1–3)	.13

Data are expressed as medians (with overall ranges).

*$P < .01$.

[a]Mann–Whitney *U* test.

IPQ, Illness Perception Questionnaire.

Table S4. TAS-20 (alexithymic personality) scores by group (scores of <8 on “Depression” subscale of HADS)

**Alexithymic characteristics**	**Fatigue group (*n* = 19)**	**Non-Fatigue group (*n* = 16)**	***P*-value[a]**
**TAS-20 total score**	47 (30–61)	43 (35–58)	0.59
Score ≧ **52,**[b] ***n* (%)**	3 (15%)	2 (12%)	1.00
Subscale			
**Difficulty identifying feelings**	14 (7–21)	9 (7–18)	0.07
**Difficulty describing feelings**	13 (5–19)	12 (6–20)	0.97
**Externally oriented thinking**	19 (14–26)	21 (15–29)	0.35

Except where noted, data are expressed as medians (with overall ranges) of scale scores.

*$P < .05$.

[a]Mann–Whitney *U* test.

[b]A total score of 52 or higher indicates possible alexithymia.

HADS, Hospital Anxiety and Depression Scale; TAS-20, Toronto Alexithymia Scale.
